# Programmable DNA repair with CRISPRa/i enhanced homology-directed repair efficiency with a single Cas9

**DOI:** 10.1038/s41421-018-0049-7

**Published:** 2018-07-24

**Authors:** Lupeng Ye, Chengkun Wang, Lingjuan Hong, Ninghe Sun, Danyang Chen, Sidi Chen, Feng Han

**Affiliations:** 1 Institute of Pharmacology and Toxicology, College of Pharmaceutical Sciences, Zhejiang University, 310058 Hangzhou, China; 2 System Biology Institute, Integrated Science & Technology Center, 850 West Campus Drive, Room 361, West Haven, CT 06516 USA; 3 Department of Genetics, Yale University School of Medicine, 333 Cedar Street, New Haven, CT 06510 USA; 4 Cardiovascular Research Center, Department of Internal Medicine, Yale University School of Medicine, 333 Cedar Street, New Haven, CT 06520 USA

## Abstract

CRISPR systems have been proven as versatile tools for site-specific genome engineering in mammalian species. During the gene editing processes, these RNA-guide nucleases introduce DNA double strand breaks (DSBs), in which non-homologous DNA end joining (NHEJ) dominates the DNA repair pathway, limiting the efficiency of homology-directed repair (HDR), the alternative pathway essential for precise gene targeting. Multiple approaches have been developed to enhance HDR, including chemical compound or RNA interference-mediated inhibition of NHEJ factors, small molecule activation of HDR enzymes, or cell cycle timed delivery of CRISPR complex. However, these approaches face multiple challenges, yet have moderate or variable effects. Here we developed a new approach that programs both NHEJ and HDR pathways with CRISPR activation and interference (CRISPRa/i) to achieve significantly enhanced HDR efficiency of CRISPR-mediated gene editing. The manipulation of NHEJ and HDR pathway components, such as CtIP, CDK1, KU70, KU80, and LIG4, was mediated by catalytically dead guide RNAs (dgRNAs), thus relying on only a single catalytically active Cas9 to perform both CRISPRa/i and precise gene editing. While reprogramming of most DNA repair factors or their combinations tested enhanced HDR efficiency, simultaneously activating *CDK1* and repressing *KU80* has the strongest effect with increased HDR rate upto an order of magnitude. Doxycycline-induced dgRNA-based CRISPRa/i programming of DNA repair enzymes, as well as viral packaging enabled flexible and tunable HDR enhancement for broader applicability in mammalian cells. Our study provides an effective, flexible, and potentially safer strategy to enhance precise genome modifications, which might broadly impact human gene editing and therapy.

## Introduction

Organisms evolved multiple mechanisms to maintain genome integrity^1,2^. As the cellular genome is constantly exposed to environmental damage, multiple DNA damage repair pathways exist to protect the genome from harmful or potentially catastrophic alterations^3^. DNA double-strand break (DSB) repair pathways are highly conserved between eukaryotes including mammalian species^4,5^. Non-homologous DNA end joining (NHEJ) and homology-directed repair (HDR) are two major DNA repair pathways that can either act in concert or antagonistic manner^6^. HDR is a pathway which uses template DNA, such as an intact sister chromosomal copy or an exogenous donor to repair the DSBs^7^, thus can robustly generate perfect repair^8^. However, HDR efficiency depends on species, cell type, and the stage of the cell cycles^8–12^. In mammalian cells, NHEJ has been considered the major pathway to repair the DNA^13^, whereas HDR is more common in the *Saccharomyces cerevisiae*^14^. NHEJ is an imperfect process, which often lead to gain or loss of a few nucleotides at each end of the breakage site^13,15^. This character can lead to subsequent deleterious genetic alteration that results in cellular malfunctioning, cancer, or aging^16^. The DNA repair enzymes KU70, KU80, and Ligase IV (LIG4) play central roles in NHEJ-mediated DNA repair, where KU70 and KU80 proteins stabilize the DNA ends and make them physical proximity to facilitate end ligation performed by *LIG4*^7^. On the other hand, proteins such as BRCA1/2, RAD50, RAD51, and various cell cycle regulators are directly involved in homology-directed repair (HDR)^9,17^, although the pathway has yet to be fully characterized.

The type II bacterial adaptive immune system, clustered regularly interspaced palindromic repeats (CRISPR)-associated protein 9 (Cas9) is a powerful genome editing tool^18–20^. The Cas9 - single guide RNA (sgRNA) complex induces site-specific DSBs, which can be repaired by either of the two main DNA repair pathways, NHEJ and HDR. The error-prone repairs by NHEJ often introduce unpredictable frame shift insertions and deletions (indels), leading to loss-of-function of target genes. In contrast, HDR can either generate perfect DNA repair or precise genome modification guided by donor templates. However, HDR is substantially less efficient compared to NHEJ^21,22^ in mammalian cells and most often restricted to S/G2 phase(s) of the cell cycle^23^. Owning to the importance of HDR in mediating precise genetic modification, extensive efforts have been made to change the balance of DNA repair pathways. However, due to the intricacy of the DNA repair pathways, the available tools to enhance HDR are still limited to a few choices with relatively small effect. Moreover, little success to date has been achieved to directly augment the HDR pathway itself. Thus, manipulation of both HDR and NHEJ using simple genetic tools might enable or strengthen a variety of genome editing applications. Recently, 14-nt or 15-nt guide RNAs have been shown to be catalytically inactive yet maintain the target-site binding capacity^24,25^. These catalytically dead guide RNAs (dgRNAs) thus can be utilized to modulate gene expression using a catalytically active Cas9^24,25^. Therefore, an active Cas9 nuclease can be repurposed to simultaneously perform genome editing and regulate gene transcription using both types of gRNAs in the same cell. We thus hypothesize that dgRNAs together with the associated CRISPR activation (CRISPRa) and interference (CRISPRi) modules can be deployed to achieve HDR enhancement using a single active Cas9.

## Results

**Fig. 1:**
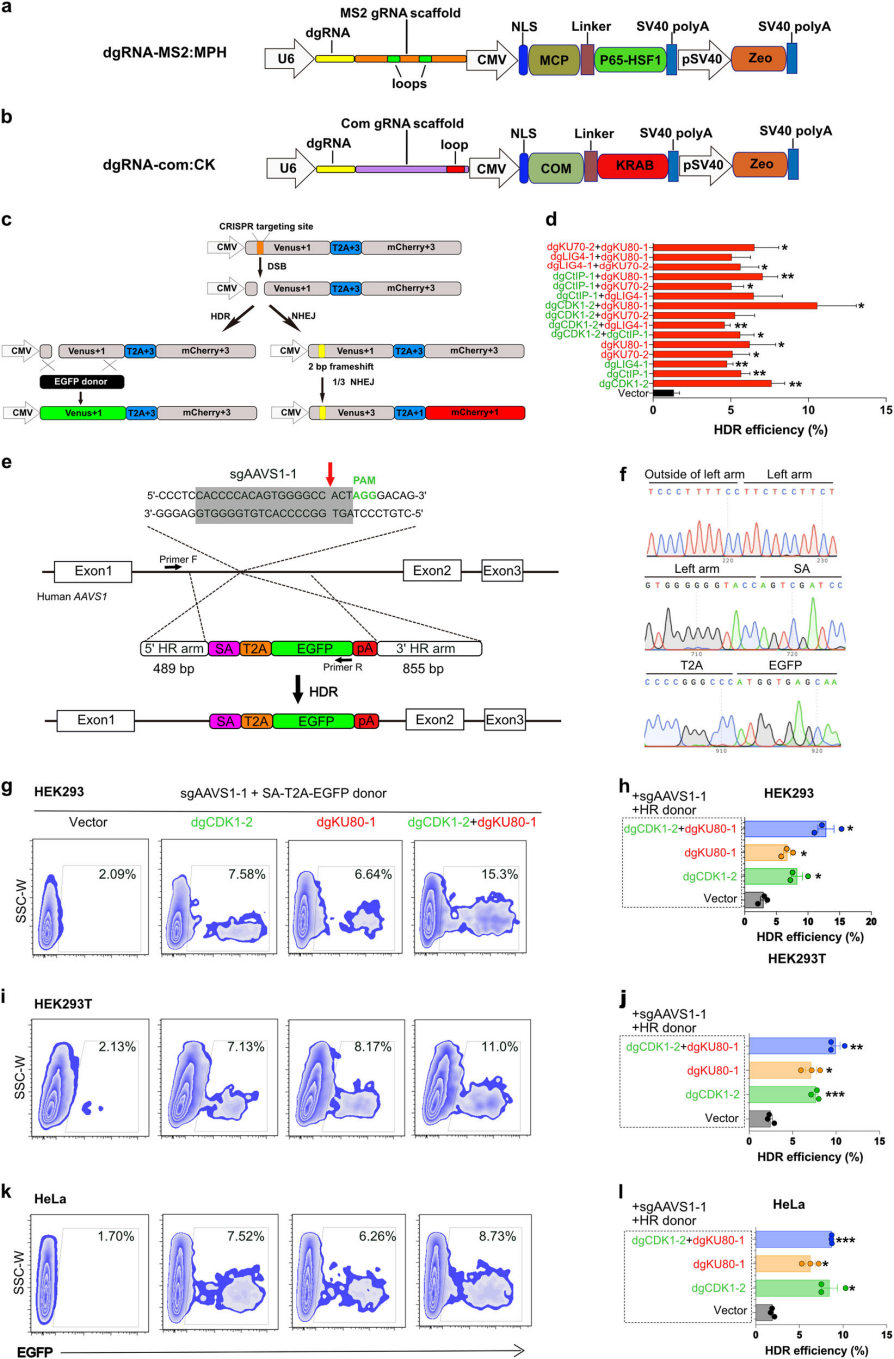
Programming key genes of HDR and NHEJ pathways enhanced HDR efficiency. **a** Diagram of dgRNA-MS2:MPH expression vector for activating key genes of HDR pathway. **b** Diagram of dgRNA-Com:CK expression vector for repressing key genes of NHEJ pathway. **c** Diagram of the TLR system. Cas9/sgRNA can induce DSBs in the target site. If DSBs are repaired by NHEJ, 3n + 2 bp frame shift indels can restore *mCherry* expression, which accounted for approximately 1/3 mutagenic NHEJ events. Alternatively, if DSBs were repaired according an intact *EGFP* template, the mutations in *bf-Venus* will be corrected, leading to Venus (EGFP variant) expression. **d** Quantitative results of HDR efficiency by programming essential components of DNA repair pathways (Vector vs. dgCDK1-2, *p* = 0.0014; Vector vs. dgCtIP-1, *p* = 0.0079; Vector vs. dgLIG4-1, *p* = 0.0031; Vector vs. dgKU70-2, *p* = 0.026; Vector vs. dgKU80-1, *p* = 0.0338; Vector vs. dgCDK1-2 + dgCtIP-1, *p* = 0.0138; Vector vs. dgCDK1-2 + dgLIG4-1, *p* = 0.0022; Vector vs. dgCDK1-2 + dgKU70-2, *p* = 0.075; Vector vs. dgCDK1-2 + dgKU80-1, *p* = 0.0493; Vector vs. dgCtIP-1 + dgLIG4-1, *p* = 0.0692; Vector vs. dgCtIP-1 + dgKU70-2, *p* = 0.0245; Vector vs. dgCtIP-1 + dgKU80-1, *p* = 0.0063; Vector vs. dgLIG4-1 + dgKU70-2, *p* = 0.0337; Vector vs. dgLIG4-1 + dgKU80-1, *p* = 0.0606; Vector vs. dgKU70-2 + dgKU80-1, *p* = 0.0299), the representative flow cytometry figures are shown in Fig. S3c. **e** Strategy for insertion of an *EGFP* reporter gene into the human *AAVS1* locus using CRISPR-Cas9 in human cells. The SA-T2A-EGFP promoterless cassette was flanked by two *AAVS1* homology arms, left arm (489 bp) and right arm (855 bp). SA, splice acceptor, T2A, a self-cleaving peptide, PA, a short polyA signal, primer F and primer R were designed for EGFP-positive events identification and sequencing. **f** Chromatogram and sequences of HDR-positive events. Partial donor sequences and adjacent genomic DNA sequence were represented. **g–l** HDR efficiency was determined in three different cell lines, HEK293 (Vector vs. dgCDK1-2, *p* = 0.0153; Vector vs. dgKU80-1, *p* = 0.0404; Vector vs. dgCDK1-2 + dgKU80-1, *p* = 0.029), HEK293T (Vector vs. dgCDK1-2, *p* = 0.0008; Vector vs. dgKU80-1, *p* = 0.0227; Vector vs. dgCDK1-2 + dgKU80-1, *p* = 0.0087) and HeLa (Vector vs. dgCDK1-2, *p* = 0.015; Vector vs. dgKU80-1, *p* = 0.0216; Vector vs. dgCDK1-2 + dgKU80-1, *p* = 0.0004). *CDK1* activation and/or *KU80* repression significantly increased HDR efficiency. These cell lines were co-transfected with SA-T2A-EGFP donor and sgAAVS1-mCherry expression vectors 24 h after dgRNA-Com:CK and/or dgRNA-MS2:MPH transfection. At day 3, the frequency of EGFP^+^ cells within mCherry^+^ population were determined using FACS. Data are showed as the mean ± SEM from three independent experiments. Significance was calculated using the Paired *t-*test. *p < 0.05, **p < 0.01, ***p < 0.001

To enhance HDR efficiency of CRISPR-mediated gene editing with clean genetic approaches that avoid the potential side effects from chemical compounds, we leverage a method that tunes the expression of DNA damage repair pathway components by dgRNA/active Cas9-mediated CRISPRa and CRISPRi (CRISPRa/i). We constructed Com binding loop into dgRNA scaffold^26^ for recruiting COM-KRAB (CK) fusion domain to repress the NHEJ-related genes, as well as MS2-binding loops into dgRNA scaffold^26,27^ for recruiting MCP - P65-HSF1 (MPH) fusion domain to activate HDR-related genes (Fig. 1a, b). With these two constructs we first tested them using an EGFP reporter system, and then two endogenous genes. The results showed robust activation and repression of both exogenous reporter genes and endogenous genes, where the EGFP’s mRNA level was significantly upregulated by dgGFP - MS2:MPH and repressed by dgGFP-Com:CK (Supplementary Fig. S1a–c), and the transcriptional level of *ASCL1* and *HBG1* were dramatically upregulated by dgRNA-MS2:MPH systems with gene-specific dgRNAs (Supplementary Fig. S1d, e). Based on the robust functions of dgRNA-MS2:MPH and dgRNA-Com:CK, we programmed the activation and repression of several key HDR and NHEJ genes, respectively. The results showed that *CDK1*, promotes efficient end resection by phosphorylating DSB resection nuclease^28^, and *CtIP*, is well-known enzyme promoting resection of DNA ends to single-stranded DNA (ssDNA) which is essential for HR^29^, transcript levels were upregulated by near three-fold, and *LIG4*, *KU70*, and *KU80* were reduced by 40–50% (Supplementary Fig. S1f–j).

We next sought to determine if *CDK1* and *CtIP* activation or *LIG4*, *KU70*, and *KU80* inhibition could enhance HDR frequency for CRISPR-mediated precise gene editing. To quantitatively determine the HDR and NHEJ outcome, we firstly generated a traffic light reporter (TLR)^30^ stable expression HEK293 cell line that also expresses Cas9 (HEK293-Cas9-TLR) (Fig. 1c). The TLR includes a nonfunctional green fluorescent reporter in which codons 53–63 were disrupted (*broken frame Venus, bf-Venus*), driven by a CMV promoter. In addition, a self-cleaving peptide T2A and a red fluorescent reporter with a 2 bp frameshift (*fs-mCherry*) were cloned closely adjacent to the *bf-Venus* (Fig. 1c). With an sgRNA targeting the 5′ region of the *bf-Venus*, Cas9 induces DSBs, which can subsequently be repaired by two major DNA repair pathways, NHEJ or HDR. NHEJ caused indels shifting the coding frame of the T2A-mCherry, where ∼1/3 of the mutagenic NHEJ events generate in-frame functional *mCherry* that could be detected in cells (Supplementary Fig. S2b). However, if an intact *EGFP* HDR donor was provided during DSB repair, the *bf-Venus* could be corrected in a precise manner that leaves the succeeding *fs-mCherry* remaining out of frame (Supplementary Fig. S2c). Thus, this TRL reporter allows us to accurately quantify HDR and NHEJ events.

Using this TLR reporter, we transfected HEK293-Cas9-TLR cell line with dgRNA-Com:CK and/or dgRNA-MS2:MPH plasmids targeting *CDK1*, *CtIP*, *LIG4*, *KU70*, and *KU80* to modulate the expression of these factors. 24 h later, cells were co-transfected with PCR *EGFP* HDR template and sgVenus-ECFP expression plasmids (Supplementary Fig. S3a). ECFP^+^ cells were gated by FACS after 48 h of transfection (Supplementary Fig. S3b), and the frequency of EGFP^+^ and mCherry^+^ cells were determined (Supplementary Fig. S3c). In the vector group, we observed 2.42% EGFP^+^ and 6.82% mCherry^+^ cells, which represented HDR-positive and NHEJ-positive events, respectively (Supplementary Fig. S3c). In contrast, the percentage of EGFP^+^ cells was dramatically increased after activating HDR-related genes by dgRNA-MS2:MPH, or repressing NHEJ-related genes by dgRNA-Com:CK, for all 15 single or combinatorial perturbations tested, with 12/15 perturbations reaching statistical significance (Fig. 1d; Supplementary Fig. S3c). Particularly, in the group of dgCDK1 - 2:MS2-MPH (dgCDK1–2) + dgKU80-1:Com-CK (dgKU80-1), 15.4% EGFP-positive cells were observed (Fig. 1d; Supplementary Fig. S3c). Of note, NHEJ has two major sub-classes of pathways, classical NHEJ (C-NHEJ) and micro-homology-mediated end-joining (MMEJ), also known as alternative end joining (Alt-EJ), which uses of 5–25 bp microhomologous sequences during broken ends alignment before the ends join, thus resulting in indels flanking the original break^31,32^. C-NHEJ is not error prone, whereas MMEJ is. Both pathways are distinct from HDR. Our assay measures the outcome of mutation acquisition, thus it measures non-classical NHEJ (MMEJ) but not C-NHEJ, where most of the repairs are not mutagenic thus is silent from the assay. Interestingly, KU70 and KU-80 repression by dgRNA showed that KU-silencing only increase template-dependent precise gene editing (HDR, as measured by Venus), but does not increase mutagenic repair (NHEJ/MMEJ, as measured by mCherry) (Supplementary Fig. S3c). Thus, suppression of KU70/KU80 may tip the balance of NHEJ (C-NHEJ and MMEJ) and HDR pathways, thereby increases the HDR efficiency. To confirm that the DSBs were repaired through HDR or NHEJ pathways, EGFP^+^/mCherry^−^, EGFP^−^/mCherry^+^ and EGFP^−^/mCherry^−^ cells were cloned and the TLR sgRNA-targeting sites were sequenced. It was observed that in EGFP^+^/mCherry^−^ clones, the *bf-Venus* gene was precisely repaired by the *EGFP* HDR donor without indels, whereas various indels were found in both EGFP^−^/mCherry^+^ and EGFP^−^/mCherry^−^ clones (Supplementary Fig. S4a–c), together confirming the HDR and NHEJ events at genomic DNA (gDNA) level. Thus, with the robust TLR system, we found that modulating HDR factors, NHEJ factors, or their combinations can significantly enhance HDR efficiency, where both programming HDR/NHEJ by CRISPRa/i and Cas9-mediated gene editing were achieved simultaneously with a single Cas9 transgene.

**Fig. 2:**
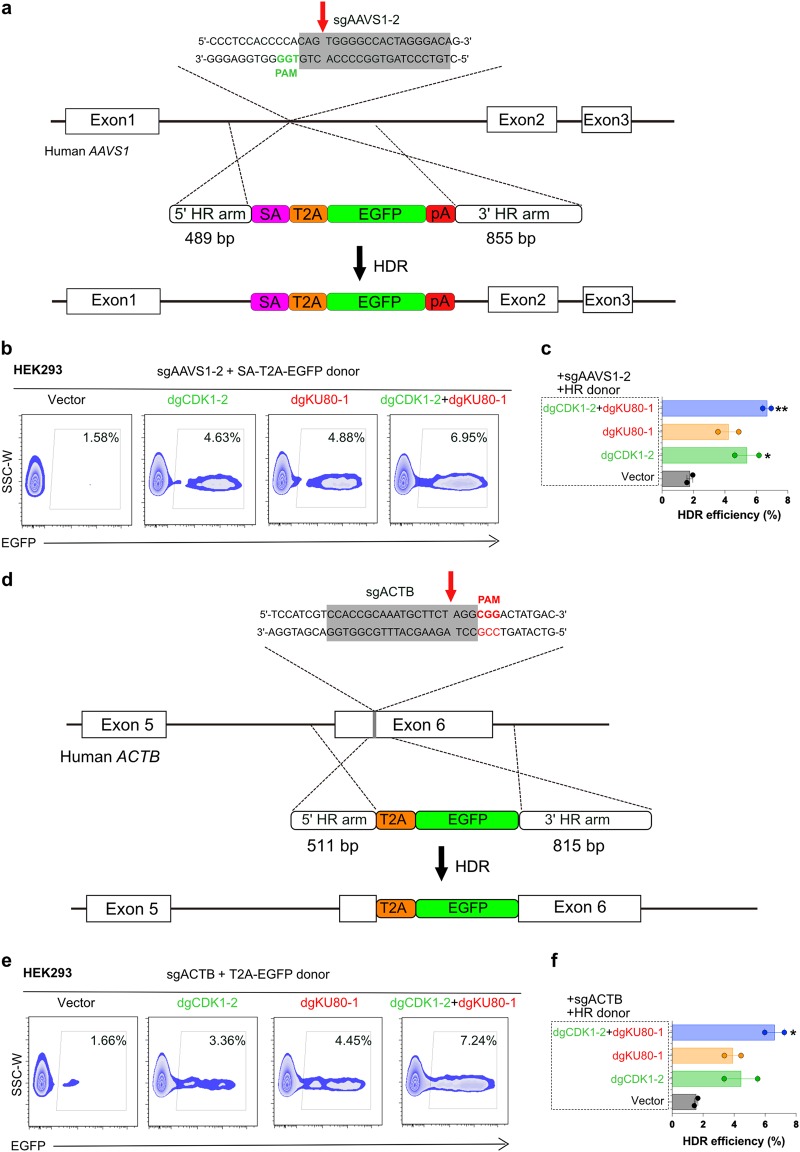
Activate *CDK1* and repress KU80 could enhance HDR efficiency in endogenous loci. **a** A scheme of insertion strategy at the human *AAVS1* locus. A new *AAVS1* targeting site was designed, sgAAVS1–2 was close to sgAAVS1-1 targeting site, but both used a same HDR donor template. **b-c** HDR efficiency at the different *AAVS1* locus (Vector vs. dgCDK1-2, *p* = 0.0443; Vector vs. dgKU80-1, *p* = 0.0699; Vector vs. dgCDK1-2 + dgKU80-1, *p* = 0.0044). **d** A scheme of insertion strategy at the human *ACTB* locus. **e-f** Flow cytometry showed that the HDR efficiency was significantly improved after activating *CDK1* and repressing *KU80* genes (Vector vs. dgCDK1-2, *p* = 0.1166; Vector vs. dgKU80-1, *p* = 0.0504; Vector vs. dgCDK1-2 + dgKU80-1, *p* = 0.0156). Significance was calculated using the Unpaired *t-*test. *p < 0.05, **p < 0.01

The dgCDK1-2 + dgKU80-1 combination has the highest enhancement of HDR efficiency as revealed by TLR experiment among all tested groups/programs. To test its effect on CRISPR-mediated gene editing at an endogenous genomic locus, we measured the precise integration of an HDR donor SA-T2A-EGFP expression cassette into the first intron of the canonical *AAVS1* locus upon Cas9/sgRNA-induced DSB (Fig. 1e). The SA-T2A-GFP was flanked by an *AAVS1* left homology arm (489 bp) and a right homology arm (855 bp), where EGFP could only be expressed when the SA-T2A-EGFP was precisely recombined into the target site (Fig. 1e). We firstly transfected dgRNA-Com:CK and/or dgRNA-MS2:MPH constructs targeting *CDK1* and *KU80* genes using the HEK293-Cas9 cell line. 24 h later, these cells were co-transfected with SA-T2A-EGFP HDR donor template and an sgAAVS1-mCherry plasmid and then analyzed by FACS 48 h after transfection. Compared to the baseline 2.09% GFP^+^ cells in the mCherry^+^ population in vector group, the fraction of GFP^+^ cells from dgCDK1-2, dgKU80-1, and dgCDK1-2 + dgKU80-1 groups were significantly increased to 7.58%, 6.64%, and 15.3%, respectively (Fig. 1g, h). Quantitative result showed that HDR efficiency was enhanced over three-fold with single factor programming and over seven-fold with dual programming on an endogenous locus *AAVS1* (Fig. 1g, h). We confirmed the results with two additional cell lines, with upto five-fold HDR enhancement in HEK293T and again five-fold in HeLa (Fig. 1i–l). We also designed another sgRNA for *AAVS1* targeting using a same HDR template (Fig. 2a), the result showed that HDR also can be significantly improved (Fig. 2b, c). In addition, we tested another gene locus, *ACTB*, activation of *CDK1* and repression of *KU80* significantly enhanced HDR upto 4–5-fold (Fig. 2d-f). Results from all those cell lines and loci showed that HDR efficiency enhancement was most dramatic in the dgCDK1–2 + dgKU80-1 combination group. We amplified, cloned, and sequenced the endogenous *AAVS1* locus and confirmed the precise integration of SA-T2A-EGFP into the anticipated target site (Fig. 1f, Supplementary Fig. S4d). Thus, in concordance with the exogenous TLR results, we observed an enhanced efficacy of precise gene targeting via HDR in the native mammalian genome.

**Fig. 3:**
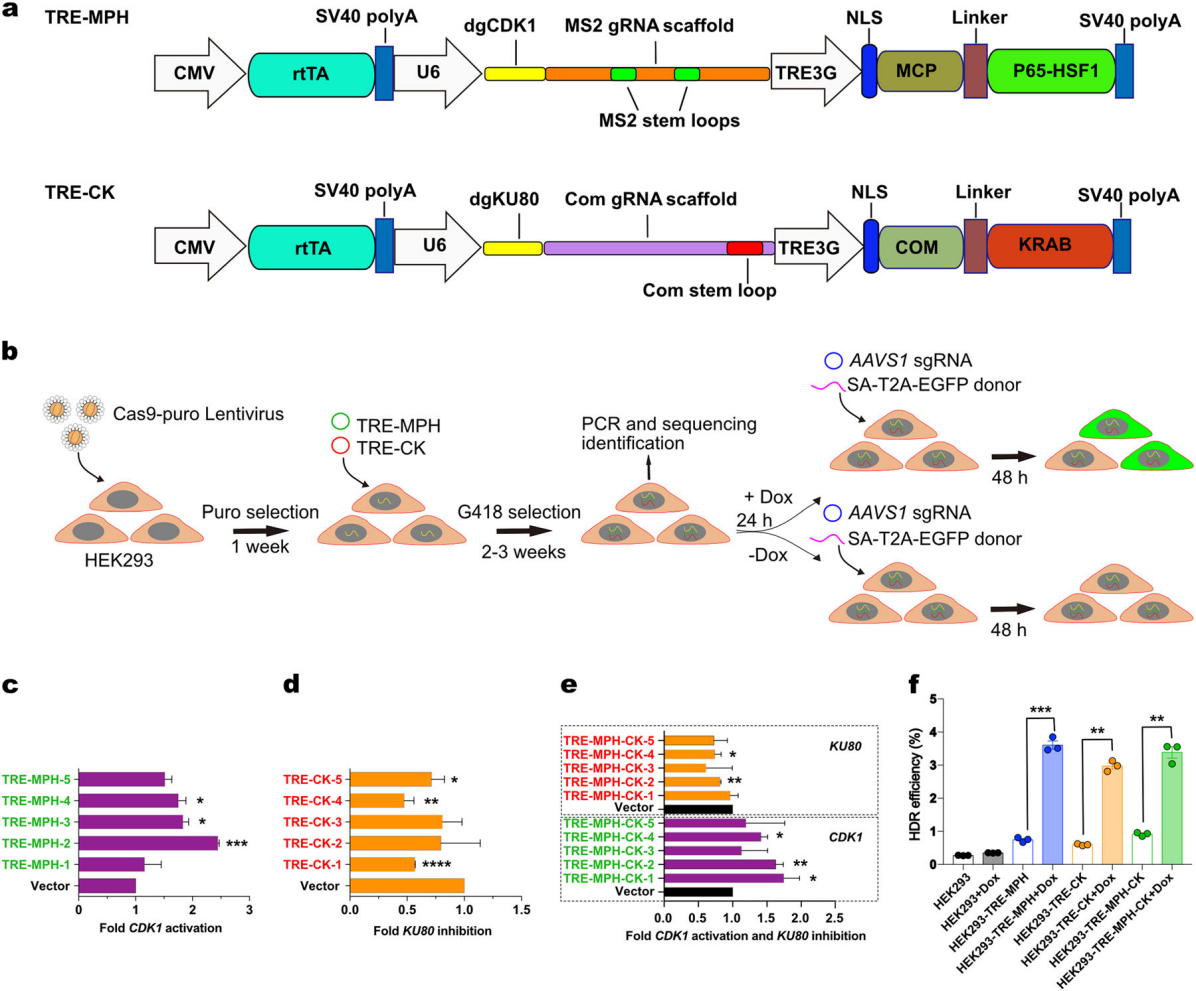
An inducible DNA repair CRISPRa/i system for enhancing HDR efficiency. **a** Diagram of TRE-MPH and TRE-CK expression vectors used to activate *CDK1* and repress *KU80*, respectively. rtTA interact with doxycycline, the complex could bind to TRE3G promoter, which then initiate the transcription of MCP-P65-HSF1 or COM-KRAB. **b** The workflow of establishing inducible HDR increasing system. Activation of *CDK1* and/or repression of *KU80* can be achieved by simply controlling the availability of doxycycline. Dox, doxycycline; Puro, puromycin. **c-e** HEK293-TRE-MPH, HEK293-TRE-CK, and HEK293-TRE-MPH-CK cell lines were obtained based on HEK293-Cas9 cell line by G418 selection. Several random clones were picked for each cell line. Although the transcriptional levels of CDK1 activation or KU80 repression can vary between clones, the clones with significant CDK1 activation and/or KU80 repression have increased HDR efficiency. The transcription level of *CDK1* and *KU80* were determined by RT-qPCR after 2 days of doxycycline treatment. **f** Quantitative analysis result of HDR efficiency (HEK293-TRE-MPH vs. HEK293-TRE-MPH + Dox, *p* = 0.001; HEK293-TRE-CK vs. HEK293-TRE-CK + Dox, *p* = 0.0011; HEK293-TRE-MPH-CK vs. HEK293-TRE-MPH-CK + Dox, *p* = 0.0032). Data was shown as the mean ± SEM from three independent experiments. Significance was calculated using the Paired *t*-test. *p < 0.05, **p < 0.01, ***p < 0.001, ****p < 0.0001

To further improve the programmability of this approach, we adapted it to additional conditional-expression modules and viral packaging system. To reduce potential side effects from constitutive activation of *CDK1* or deficiency of *KU80*, we utilized a Tet-On system inducible by doxycycline (Dox) to control the expression of CRISPRa and CRISPRi effectors, MPH and CK, respectively. We constructed two vectors, TRE-MPH and TRE-CK (Fig. 3a). Both vectors contain a CMV-rtTA expression cassette, when cells are treated with Dox, the rtTA protein will specifically bind to TRE3G promoter and thereby initiate the transcription of MPH or CK downstream (Fig. 3a), which will be reversibly turned off upon Dox removal. We transfected these plasmids into HEK293-Cas9 individually and in combination, then followed by G418 selection and cell cloning to obtain TRE-MPH, TRE-CK, and TRE-MPH-CK cell lines (Fig. 3b). By qRT-PCR, we determined that *CDK1* and *KU80* will be significantly activated or repressed, respectively, in a select set of stable cell lines (Fig. 3c, d). We chose TRE-MPH-2 and TRE-CK-4 based on their best potency of Dox-induced *CDK1* activation and *KU80* repression for the subsequent endogenous HDR experiment. Three different cell lines were treated with Dox for 24 h, then the SA-T2A-EGFP HDR donor for *AAVS1* locus and sgAAVS1-mCherry plasmid were co-transfected. After 48 h of transfection, EGFP^+^ cells in mCherry^+^ population were quantified by FACS, which revealed that upon Dox treatment, the percentages of EGFP^+^ cells significantly increased in all three groups as compared to control (Supplementary Fig. S5a), and without any side effects for Dox (Supplementary Fig. S5b), albeit interesting with a similar four-fold enhancement possibly due to the capacity of Dox-inducible gene expression. Of note, although the transcriptional levels of CDK1 activation or KU80 repression can vary between clones, the clones with significant CDK1 activation and/or KU80 repression have increased HDR efficiency. This data suggests that the CRISPRa/i DNA repair programming can be used in conjunction of inducible expression system to allow further control of HDR enhancement.

**Fig. 4:**
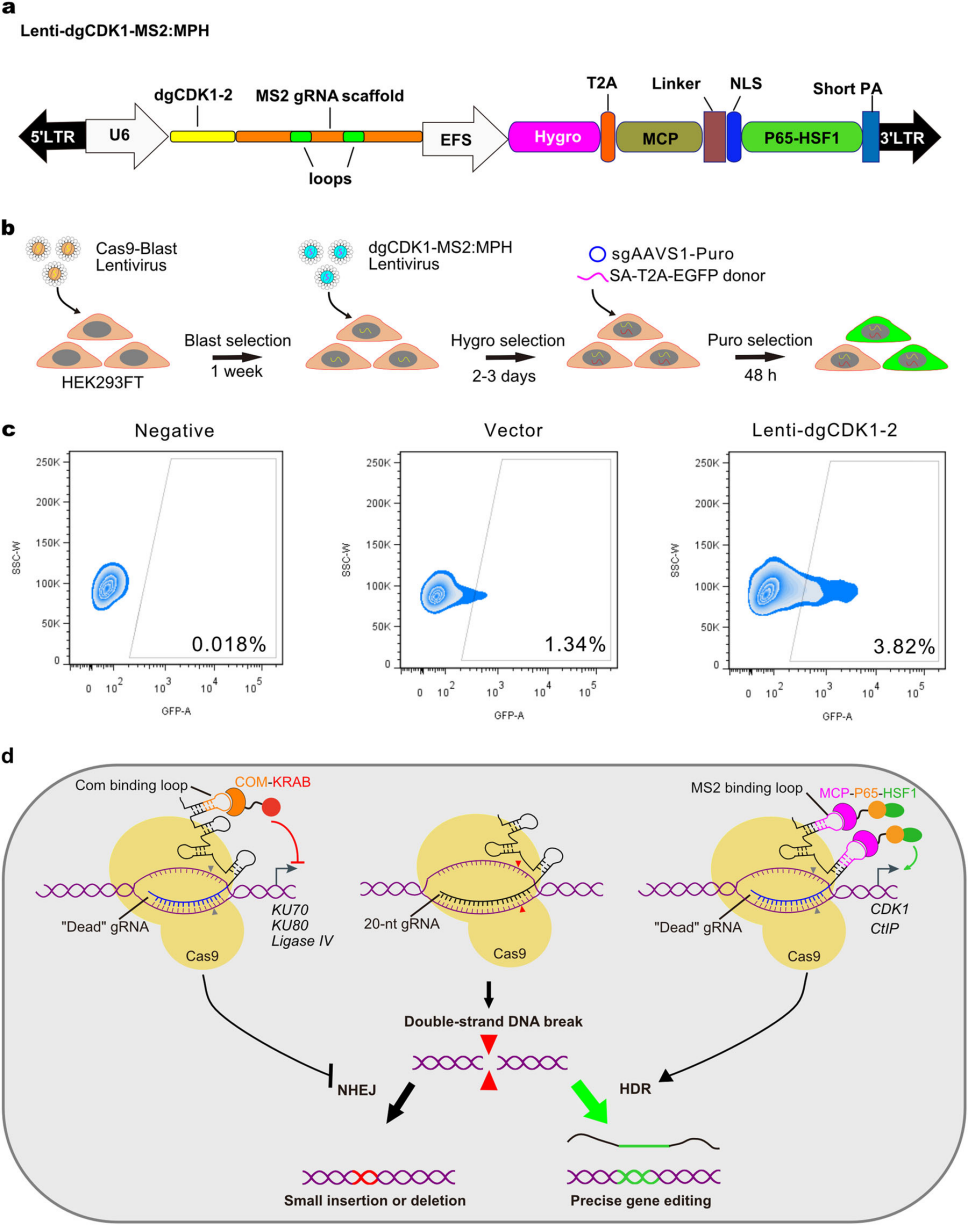
Packaging the DNA repair CRISPRa/i system with lentivirus for enhancement of HDR efficiency with viral delivery. **a** The *CDK1* activation plasmid was reconstructed into lentivirus backbone. Hygro, hygromycin. **b** HEK239FT cell was transduced with Cas9-Blast lentivirus to establish Cas9 constitutively expressed cell-line. Then the HEK239FT-Cas9 cell-line was transduced with dgCDK1-MS2:MPH lentivirus, followed by 2-3 days hygromycin selection. Finally, cells were transfected with sgAAVS1-Puro plasmid and SA-T2A-EGFP HR donor. The flow cytometry analysis was performed after 2 days' puromycin selection. Blast, blasticidin; Puro, puromycin. **c** The flow cytometry results demonstrated that HDR efficiency was significantly increased as compared with the vector group. **d** Schematic diagram representing the central idea of this study: with a single Cas9, through combinatorial usage of sgRNA and dgRNA for gene editing and CRISPRa/i on HDR/NHEJ machinery, HDR efficiency enhancement was achieved.

Finally, we adopt the usage of lentiviral system for stable integration of constructs for CRISPRa of DNA repair factors (Fig. 4a). We generated lentivirus-integrated cell lines expressing dgCDK1-MS2:MPH, and repeated the endogenous AAVS1 targeting experiment with introduction of HDR donor and sgAAVS1-Puro by transfection (Fig. 4b). Consistent with results above, FACS analysis again showed significant enhancement of HDR efficiency (Fig. 4c), indicating the adaptability of this DNA repair programming-mediated HDR enhancement system to viral delivery vehicles. In conclusion, our data together showed that CRISPRa/i-mediated activation and inhibition of key genes related to DNA damage repair pathways is an effective way to increase the efficiency of HDR for precise genome editing in mammalian cells. With the activation of CDK1 by dgRNA-MS2:MPH and/or repression of KU80 by dgRNA-Com:CK, the HDR efficiency can be enhanced by 4–8 fold. In this system, through combinatorial usage of sgRNA and dgRNA for different purposes, we achieved the genome editing, gene activation, and repression simultaneously simply with a single Cas9 transgene (Fig. 4d).

## Discussion

Our approach provides a new way for HDR enhancement compared to other approaches. LIG4 inhibitor SCR7, which was initially defined as an anti-cancer agent^33^, was used to block the NHEJ pathway and improved the efficiency of Cas9-mediated HDR^34,35^. High-throughput screening identified two small molecule compounds (L755507 and Brefeldin A) that can enhance CRISPR-mediated HDR frequency by three-fold and two-fold, respectively^36^. However, these chemicals can have vast side effects or toxicity, and face availability and delivery challenges for in vivo genetic manipulations. SCR7, L755507, and Brefeldin A have insignificant effect on improvement of HDR efficiency at endogenous *CTNNB1* and *PRDM14* loci in human cells^37^. SCR7 or L755507 were shown to have minimal effects on HDR improvement of Cas9-mediated or TALEN-mediated knock-in in rabbit, HEK293 or human fibroblast cells^38,39^, possibly due to different modes of action or different cellular availability of these small molecules across various biological settings. Notably, severe cell death was observed after SCR7 treatment on pluripotent stem cells^38^. In addition, RS-1, a stimulator of human HDR protein RAD51^38,39^, and Ad4E1B-E4orf6, which were reported as an inhibitor of NHEJ pathway^35^, also been used to increase HDR efficiency and achieved a 3–6-fold effect. Interestingly, a recent study suggested that these small compounds could decrease HDR in certain settings^37^. Therefore, small molecular compounds are effective approaches for improving HDR efficiency, while their stability, bioavailability, side effects, toxicity, and modes of action might vary across different settings.

Another strategy to enhance the HDR efficiency is restriction of cell cycle to S and G2 phases. Timed delivery of Cas9-guide RNA ribonucleoprotein (RNP) complexes into cell cycle arrested cells using aphidicolin or nocodazole inhibitors can significantly increase HDR rates in HEK293T cells^40^. Similar alternative approaches have been used to boost HDR events by fusing a peptide from human Geminin with Cas9, making the fusion protein enriched during S, G2, and M phases but lower in G1^41,42^, because cell-cycle-controlling E3 ubiquitin ligase, APC/Cdh1, is active in the late M and G1, which can ubiquitinate Geminin then leading to their degradation^43,44^. Cas9-hGem increased the rate of HDR up to 1.87-fold compared to wild-type Cas9^41^. These approaches are challenging to be carried out in vivo, as cell cycles in mammalian organs are delicately controlled in a temporal and spatial manner.

Our approach is versatile and flexible, with active-Cas9-dgRNA mediating CRISPRa/i programming of DNA repair machinery, where the active Cas9 can still perform its function of generating DSB for HDR-mediated precise gene editing. These components can join force with an armamentarium of other genetic tools, such as inducible gene expression modules via simple genetic engineering. Furthermore, the CRISPRa/i constructs can be packaged into viral vectors for efficient delivery into a large repertoire of cell types. For in vivo manipulation, the construction size of CRISPRa/i is slightly larger than that traditionally used for Cas9-based HDR, and two AAV systems^45^ can be used for simultaneously delivering activation or/and repression components and the HDR donor template. Because DNA damage repair pathways are highly conserved, especially in mammalian species and cell types^3,14^. It has been demonstrated that CRISPR-mediated HDR and NHEJ occur in various cultured cell types including ES cells^19^, primary HSC^46^, and live animals in vivo^47,48^. Therefore, the dgRNA-based CRISPRa/CRISPRi HDR enhancement system might be worthwhile to be tested in cell types of higher clinical interest, such as ES cells and HSCs. Finally, this is a genetic approach of HDR enhancement thus can be easily adapted for in vivo settings in time-specific and tissue-specific manner, which is essential for the application of gene therapy.

## Materials and methods

### Generation of activation and repression plasmids

The activation plasmid dgRNA-MS2:MPH contains a U6 promoter, an MS2 gRNA scaffold, a CMV promoter, and a MCP-P65-HSF1 complex. The repression plasmid dgRNA-Com:CK consists of a U6 promoter, a Com gRNA scaffold, a CMV promoter, and a COM-KRAB complex. All key DNA fragments in these plasmids were synthesized by GENEWIZ or IDT, then cloned into pUC57, or lentiviral plasmids using general molecular cloning and Gibson assembly (NEB). dgRNAs (14-nt or 15-nt) (Supplementary Table S1) were designed to target the first 200 bp upstream of each TSS^27^. Five dgRNAs were designed to target each gene. TRE-MPH and TRE-CK were constructed based on dgRNA-MS2:MPH and dgRNA-Com:CK by inserting CMV-rtTA cassette and replacing CMV promoter, which drive MPH or CK expression with a TRE3G inducible promoter. For establishment of TRE-MPH, TRE-CK, and TRE-MPH-CK cell lines, we firstly transduced HEK293 cells with Cas9-expressing lentivirus to establish a constitutive Cas9 expression cell line, then transfected with TRE-MPH and/or TRE-CK plasmids followed by G418 selection and PCR identification.

### Traffic light reporter (TLR) plasmid construction

TLR construct was assembled with a nonfunctional *EGFP* variant (*bf-Venus*), where codons 53–63 were disrupted, a T2A peptide, and a red fluorescent gene that has a 2-bp shifted reading frame (*fs-mCherry*)^30^. The expression cassette of Venus-T2A-mCherry was cloned in between the CMV promoter and SV40 poly (A) signal. The CRISPR-targeting site was designed at *bf-Venus* disrupted region. As Cas9 specifically induced DSBs, if DSBs were repaired by NHEJ pathway, ∼1/3 of repaired events generate in-frame functional *mCherry*. Alternatively, if DSBs were repaired by the *EGFP* HDR donor to generate intact *Venus*, the disrupted region of *bf-Venus* would be corrected that leaves *fs-mCherry* remaining out of frame.

### Cell culture and transient transfection

HEK293, HEK293T, HEK293FT, and HeLa cell lines were used in this study. Cells were maintained in complete media (DMEM (Invitrogen/Thermofisher) with 10% FBS (Gibco), penicillin (100 U/mL), and streptomycin (100 μg/mL) (Life Technologies/Thermofisher)) in 37 °C, 5% CO_2_ incubators. Before performing the activation and repression experiments, we generated Cas9-stable expressed cell lines, HEK293-Cas9, HEK293T-Cas9, HEK293FT-Cas9, and HeLa-Cas9, either by stable integration or by transduction with Cas9 lentivirus (Cas9-Puro or Cas9-Blast), followed by puromycin or blasticidin selection. All the following activation and repression experiments were based on Cas9 stable-expression cell lines. The cells were cultured in 24-well plates (Corning) in complete media and transfected with plasmids using Lipofectamine 3000 (Invitrogen) in accordance with the manufacturer’s instructions. In brief, 100,000 cells/well were seeded into 24-well plates 12 h before transfection. 600 ng of plasmid encoding dgRNA-MS2:MPH or dgRNA-Com:CK were transfected with 1 μL Lipofectamine 3000 and 1 μL P3000 reagent in Opti-MEM (Invitrogen). Cells were trypsinized and re-seeded into another 24-well plate after 24 h of transfection. After 12 h of plating, cells were transfected with a 1:1 mass ratio of sgRNA plasmid and PCR HR donor. 600 ng total plasmid per well was transfected with 1 μL Lipofectamine 3000 and 1 μL P3000 reagent. Puromycin (0.5 μg/mL), Zeocin (200 μg/mL), or Blasticidin (5 μg/mL) were added after 24 h of transfection. Media was changed per 24 h with fresh pre-warmed selection media. For Tet-On induction of gene expression, cells were treated 2 days with Dox at 1 μg/mL.

### Lentivirus production and transduction

Briefly, HEK293FT cells (ThermoFisher) were cultured in DMEM (Invitrogen) + 10% FBS (Sigma) media and seeded in 15-cm dishes before transfection. When cell confluent reached 80–90%, the media would be replaced by 13 mL pre-warmed OptiMEM (Invitrogen). For transfection of each dish, 20 μg transfer plasmids, 15 μg psPAX2 (Addgene 12260), 10 μg pMD 2.G (Addgene 12259), and 130 μL PEI were added into 434 μL OptiMEM, briefly vortexed, and incubated at room temperature for 10 min before being added to the 13 mL OptiMEM. The 13 mL OptiMEM would be replaced with pre-warmed 10% FBS in DMEM. Lentivirus supernatant was harvested 48 h after media change and aliquoted, and stored at −80 °C in a freezer. For Cas9-Puro or Cas9-Blast transduction, HEK293, HEK293T, HEK293FT, and HeLa cell lines were transduced with Cas9-Puro or Cas9-Blast lentivirus and supplemented with 2 μL of 2 mg/mL polybrene (Millipore) in six-well plates. The puromycin (0.5 μg/mL) or blasticidin (5 μg/mL) selection was performed for 7 days after lentivirus transduction. For dgCDK1-MS2-MPH lentivirus transduction of HEK293FT-Cas9 cell line, hygromycin (200 μg/mL) selection was performed for 2–3 days.

### RT-qPCR

Cells were collected and lysed using TRIzol (Invitrogen) after 48 h of drugs treatment. Total RNA was isolated using the RNAiso Plus (Takara). cDNA synthesis was performed using the Advantage RT-for-PCR kit (Takara). RNA level was quantified by qPCR using SYBR Fast qPCR Mix (Takara) in 20 μL reaction, qPCR was carried out using the CFX96 Touch Real-Time PCR Detection System (Bio-Rad). We use the melt curve to confirm the specificity of primers. mRNA relative expression level was normalized to *GAPDH* expression by the ΔΔCt method.

### Confocal fluorescence imaging

Before performing confocal fluorescence imaging, transfected cells were trypsinized and re-seeded on glass cover slips overnight. After aspirating the medium, cells were treated with 4% formaldehyde/PBS for 15 min for fixation, where their nuclei were stained with DAPI (CST) in PBS. EGFP or mCherry fluorescence was visualized by a confocal microscope (Zeiss LSM 800). Confocal data were analyzed by Image J software (NIH, Bethesda, MD, USA).

### Flow cytometry analysis

Flow cytometric (or FACS) assays were used to evaluate the percentage of EGFP-positive or mCherry-positive cells. Briefly, HEK293-Cas9, HEK293T-Cas9, HEK293FT-Cas9, and HeLa-Cas9 cells were transfected with sgRNA plasmid and HR donor, then cultured for 72 h. The cells were digested by Trypsin without EDTA, followed by brief centrifugation and resuspension in PBS, then determining the cell density and diluting to 1 × 10^6^ cell/mL. Finally, these samples were analyzed using a BD Fortessa or BD FACSAria flow cytometer within one hour.

### Genomic DNA isolation and DNA sequencing

The transfected cells were lysed and gDNA was extracted using the DNeasy Tissue Kit (Qiagen) following the manufacturer’s instruction. For HDR-positive events identification, PCR was performed using PrimeSTAR HS DNA Polymerase (Takara) with sequence-specific primers (Supplementary Table S3) using the condition: 95 °C for 4 min; 35 cycles of 95 °C for 20 s, 60 °C for 30 s, 72 °C for 1 min; 72 °C for 2 min for the final extension. PCR products were run on 1.5% agarose gel (Biowest). The specific DNA bands were recovered using AxyPrep DNA Gel Extraction Kit (Axygen). Purified PCR products were cloned into the pMD-19 T vector (Takara) according to the standard manufacturer’s instructions or directly sequenced by specific primers. Plasmid mini-preperations were performed using the AxyPrep Plasmid Miniprep Kit (Axygen), and midi-preparations were performed using QIAGEN Plasmid *Plus* Midi Kit (Qiagen). All sequencing confirmations were carried out using Sanger sequencing.

### Cell cycle analysis

Cells were harvested after CRISPR/dgRNAs activation or/and repression for 72 h, and prepared single cell suspension in PBS with 0.1% BSA. Washed and spinned cells at 400×*g* for 5 min then being resuspended with precooled 70% ethanol, fixed cells at 4 °C overnight. Washed cells in PBS, spinned cells at 500×*g* for 5 min then being resuspended in 500 μL PBS containing 50 μg/mL Propidium Iodide (PI), 100 μg/mL RNase, and 0.2% Triton X-100, incubation at 4 °C for 30 min. Before flow cytometry analysis, cells were passed through 40 μm cell strainer to remove cell aggregates.

### CCK-8 assay

Cell viability was measured using a Cell Counting Kit-8 (CCK-8) assay (Dojindo; CK04). The transfected cells (24 h after transfection) were seeded in a 96-well plate at a density of 2.5–5 × 10^3^ cells. Cells were incubated for 1 h with 110 μL complete DMEM media with 10 μL CCK-8 reagent for 24 h. Cell viability detection was performed by measuring the optical absorbance at 450 nm by using a multimode reader (Beckman Coulter; DTX880).

### Reagent, code, and data availability

All relevant plasmids and sequencing data will be publicly deposited upon publication. No custom codes were used in this study.

### Blinding statement

Investigators were not blinded for data collection or analysis. Most experiments were repeated at least three times to ensure reproducibility.

## Electronic supplementary material

Supplementary Information
