# Supplementary material for: Programmable DNA repair with CRISPRa/i enhanced homology-directed repair efficiency with a single Cas9
Source: Cell Discov. 2018 Jul 24;4:46. doi: 10.1038/s41421-018-0049-7 (PMC6056518; doi:10.1038/s41421-018-0049-7)
Supplement: Supplementary file 1 — Supplementary Information [file 41421_2018_49_MOESM1_ESM.pdf]

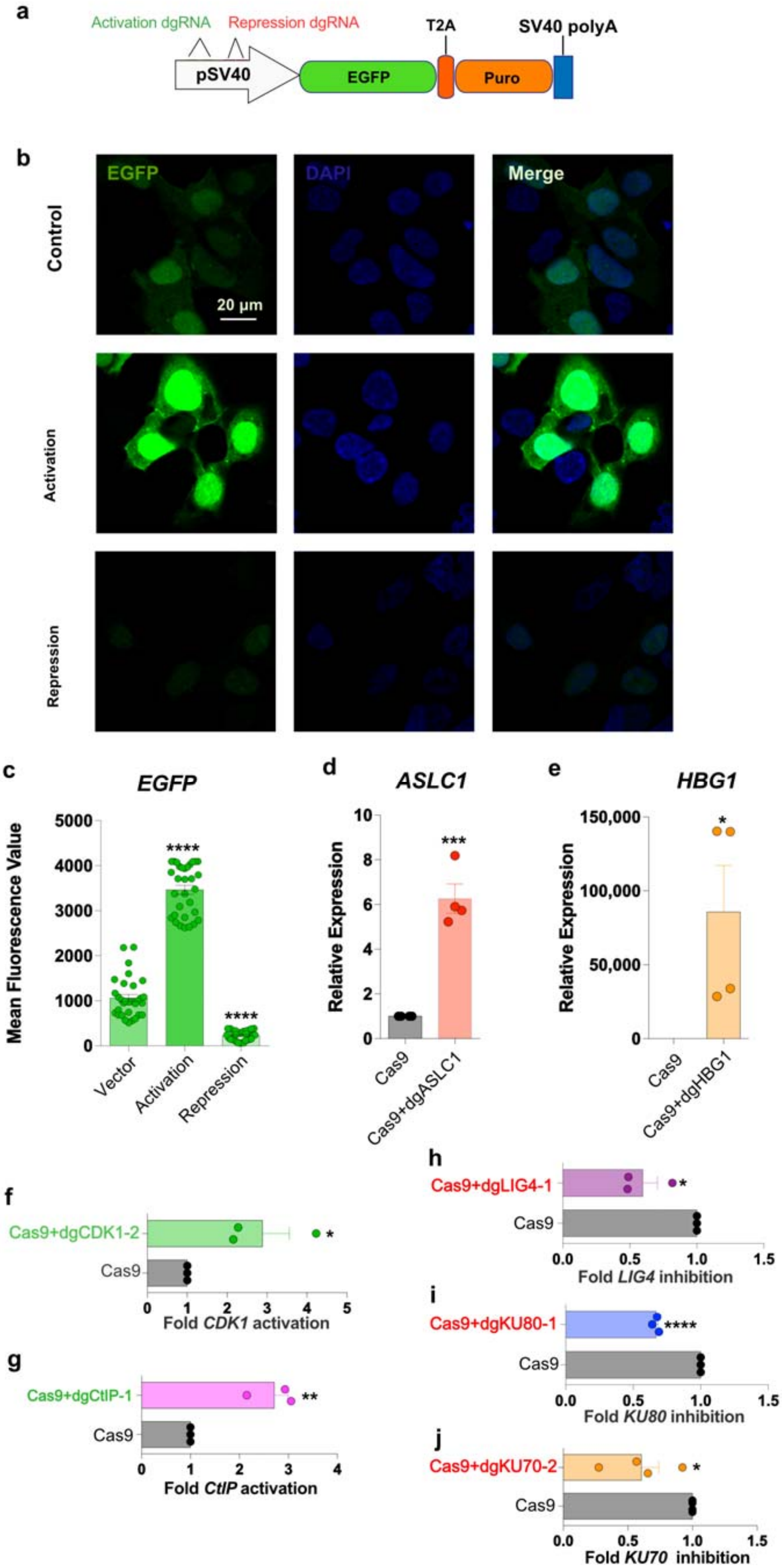

a
Activation dgRNA
Repression dgRNA
T2A
SV40 polyA
pSV40
EGFP
Puro
b
EGFP
DAPI
Merge
Control
Activation
Repression
20 μm
c
EGFP
Mean Fluorescence Value
Vector
Activation
Repression
d
ASLC1
Relative Expression
Cas9
Cas9+dgASLC1
e
HBG1
Relative Expression
Cas9
Cas9+dgHBG1
f
Cas9+dgCDK1-2
Cas9
Fold CDK1 activation
g
Cas9+dgCtIP-1
Cas9
Fold CtIP activation
h
Cas9+dgLIG4-1
Cas9
Fold LIG4 inhibition
i
Cas9+dgKU80-1
Cas9
Fold KU80 inhibition
j
Cas9+dgKU70-2
Cas9
Fold KU70 inhibition

Supplementary Fig. S1 Functional test of the dgRNA-Com:CK and dgRNA-MS2:MPH expression vectors. (a) Schematics of plasmids used for testing dgRNA-Com:CK and dgRNA-MS2:MPH systems. **(b)** Confocal analysis of dgRNA-Com:CK and dgRNA-MS2:MPH systems in HEK293 cells. HEK293 cells were transfected with pSV40-EGFP plasmid. One day later, the dgRNA-Com:CK or dgRNA-MS2:MPH expression vector targeting SV40 promoter (pSV40) was transfected. After 2 days, the fluorescence intensity was assessed using confocal microscopy. **(c)** Quantitative fluorescence intensity of EGFP after activation and repression (Vector vs Activation, $p < 0.0001$; Vector vs Repression, $p < 0.0001$). The activation efficiency of *ASLC1* (Cas9 vs Cas9 + dgASLC1, $p = 0.0002$) **(d)** and *HBG1* (Cas9 vs Cas9 + dgHBG1, $p = 0.0344$) **(e)** in HEK293 cells using dgRNA-MS2:MPH expression vector targeting *ASLC1*or *HBG1* promoter region. Three days later, total RNA was extracted and the gene transcriptional level was determined by RT-qPCR. **(f-j)** The activation or suppression efficiency of essential genes related to DNA repair. Five dgRNAs were designed for each gene to screen the best dgRNA for *CDK1* (Cas9 vs Cas9 + dgCDK1-2, $p = 0.0485$) and *CtIP* (Cas9 vs Cas9 + dgCtIP-1, $p = 0.0038$) activation and *LIG4* (Cas9 vs Cas9 + dgLIG4-1, $p = 0.0216$), *KU80* (Cas9 vs Cas9 + dgKU80-1, $p < 0.0001$) and *KU70* (Cas9 vs Cas9 + dgKU70-2, $p = 0.0254$) repression. Data was represented as the mean ± SEM from three independent experiments. Significance was calculated using the Paired t test. * $P < 0.05$, ** $P < 0.01$, *** $P < 0.001$, **** $P < 0.0001$.

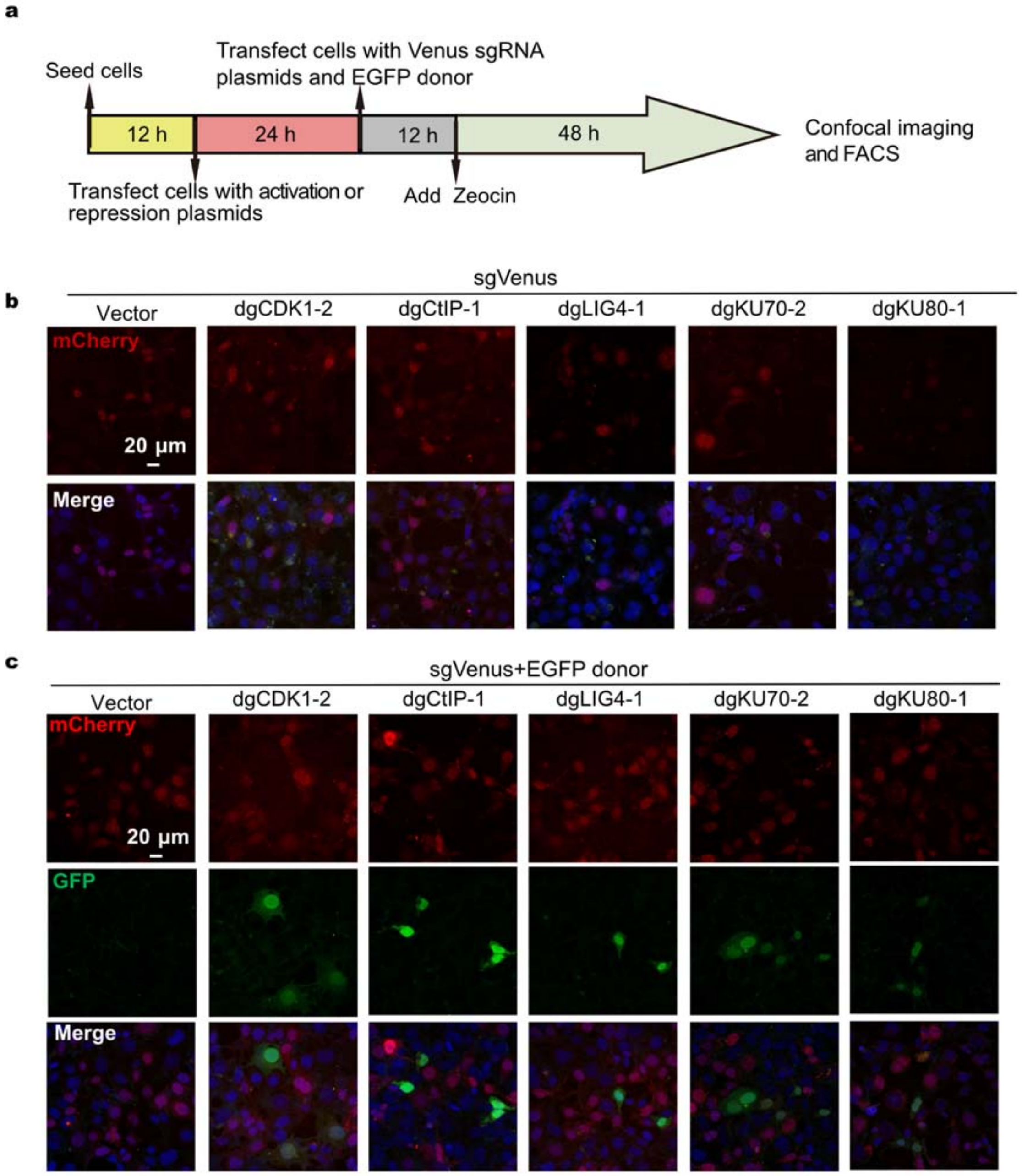

Supplementary Fig. S2 Using the TLR reporter to evaluate HDR efficiency enhancement and confocal microscopy analysis. (a) The strategy used in this experiment. Firstly, cells were transfected with dgRNA-Com:CK or dgRNA-MS2:MPH vector to active or repressing targeted gene. After 1 day, these cells were co-transfected with EGFP HR donor and sgVenus vector. 2.5 days later, the samples were analyzed by confocal microscopy or flow cytometry. **(b)** HEK293-Cas9-TLR cells co-transfected with dgRNA-Com:CK or dgRNA-MS2:MPH plasmids and sgVenus vector. After 2 days, mCherry^{+} cells were analyzed by confocal microscopy. **(c)** HEK293-Cas9-TLR cells were co-transfected with intact EGFP PCR repair template and sgVenus plasmids after dgRNA-Com:CK or dgRNA-MS2:MPH plasmid transfection. 3 days later, samples were analyzed by confocal microscopy. The ratio of HDR-positive events was significantly increased after programming DNA repair pathways.

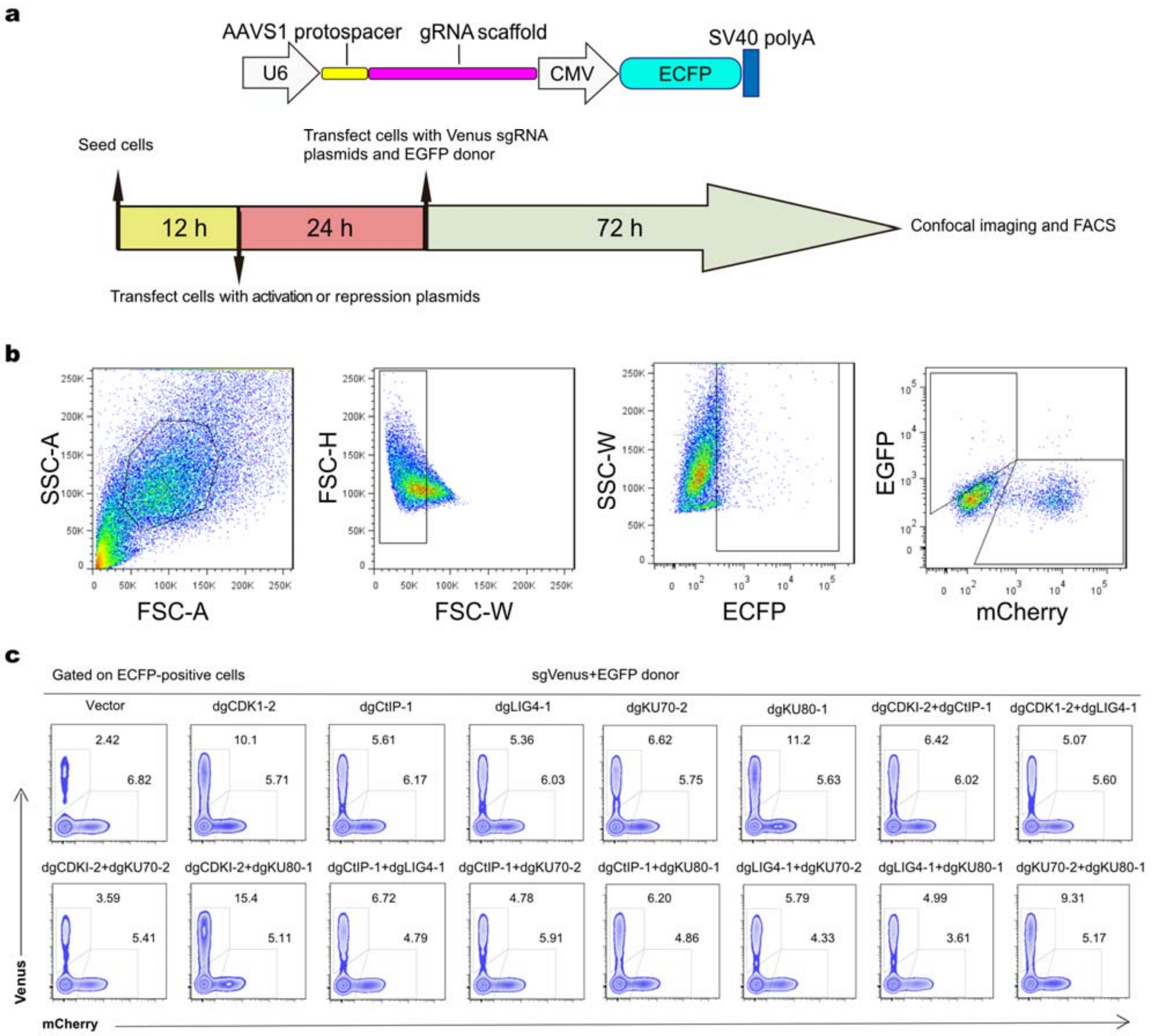

Supplementary Fig. S3 NHEJ and HDR efficiency evaluation by the TLR system using FACS. (a) The AAVS1 sgRNA plasmid schematics (upper) and the workflow of this experiment (lower). **(b)** Flow cytometry gating settings for TRL analysis of HDR and NHEJ. **(c)** HEK293-Cas9-TLR cell line was first transfected with dgRNA-MS2:MPH and/or dgRNA-Com:CK plasmids; 24 h later, these cells were co-transfected with intact EGFP PCR repair templates and sgVenus-ECFP plasmids. FACS analysis was performed after 72 h of transfection, where $ECFP^+$ cells were positively gated for transfection, and the percentage of $Venus^+$ (HDR) cells and $mCherry^+$ (NHEJ) cells were determined.

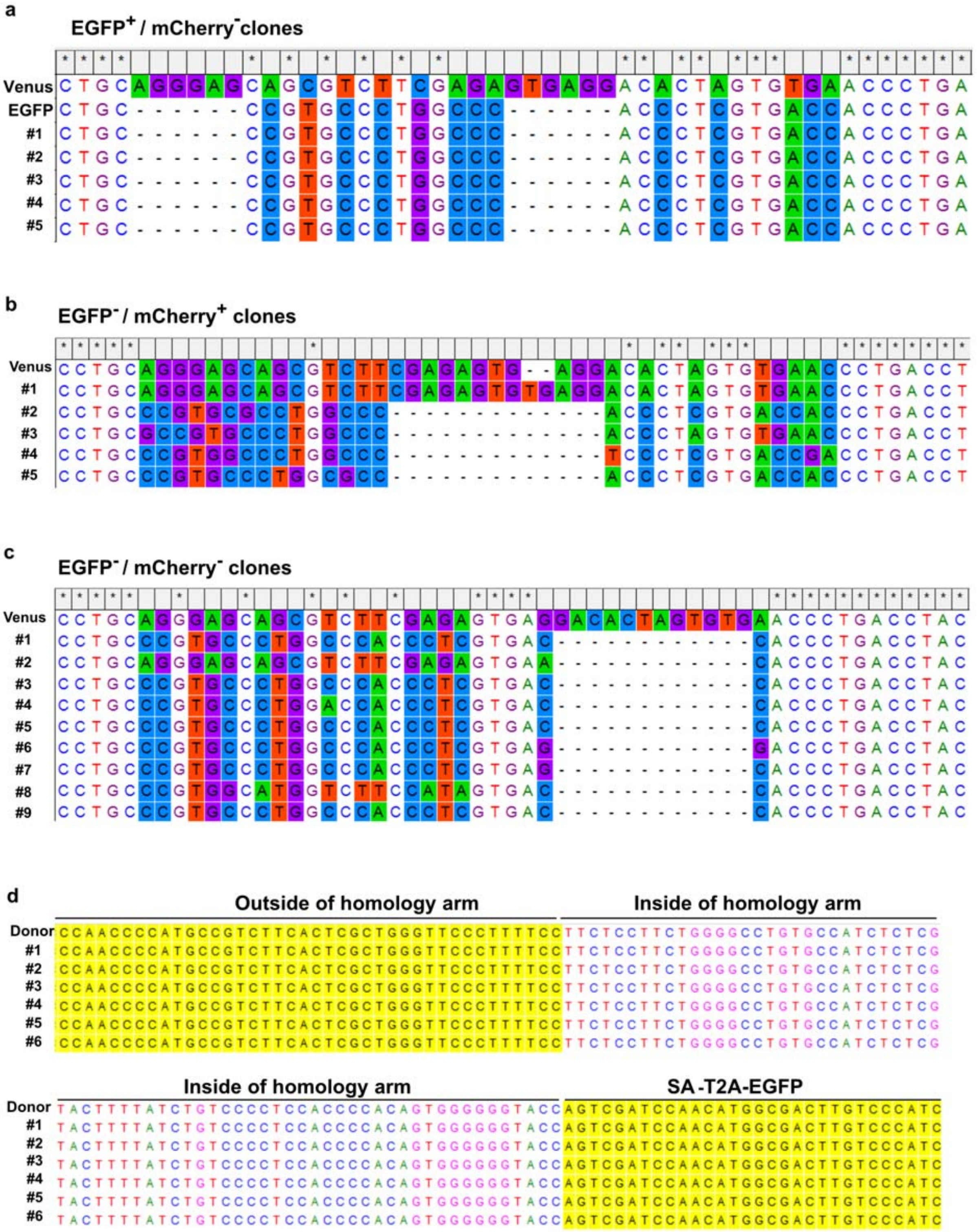

Supplementary Fig S4 Sequencing confirmation of HDR- and NHEJ-positive events and exogenous gene into the endogenous *AAVS1* locus. (a) GFP^{+}/mCherry^{-}, GFP^{-}/mCherry^{+} and GFP^{-}/mCherry^{-} individual clones were randomly picked, cultured, PCR and Sanger sequenced. Sequences from multiple clones were shown. **(b)** Sequencing confirmation of EGFP^{+} cell clones to make sure SA-T2A-EGFP was precisely integrated into *AAVS1* locus.

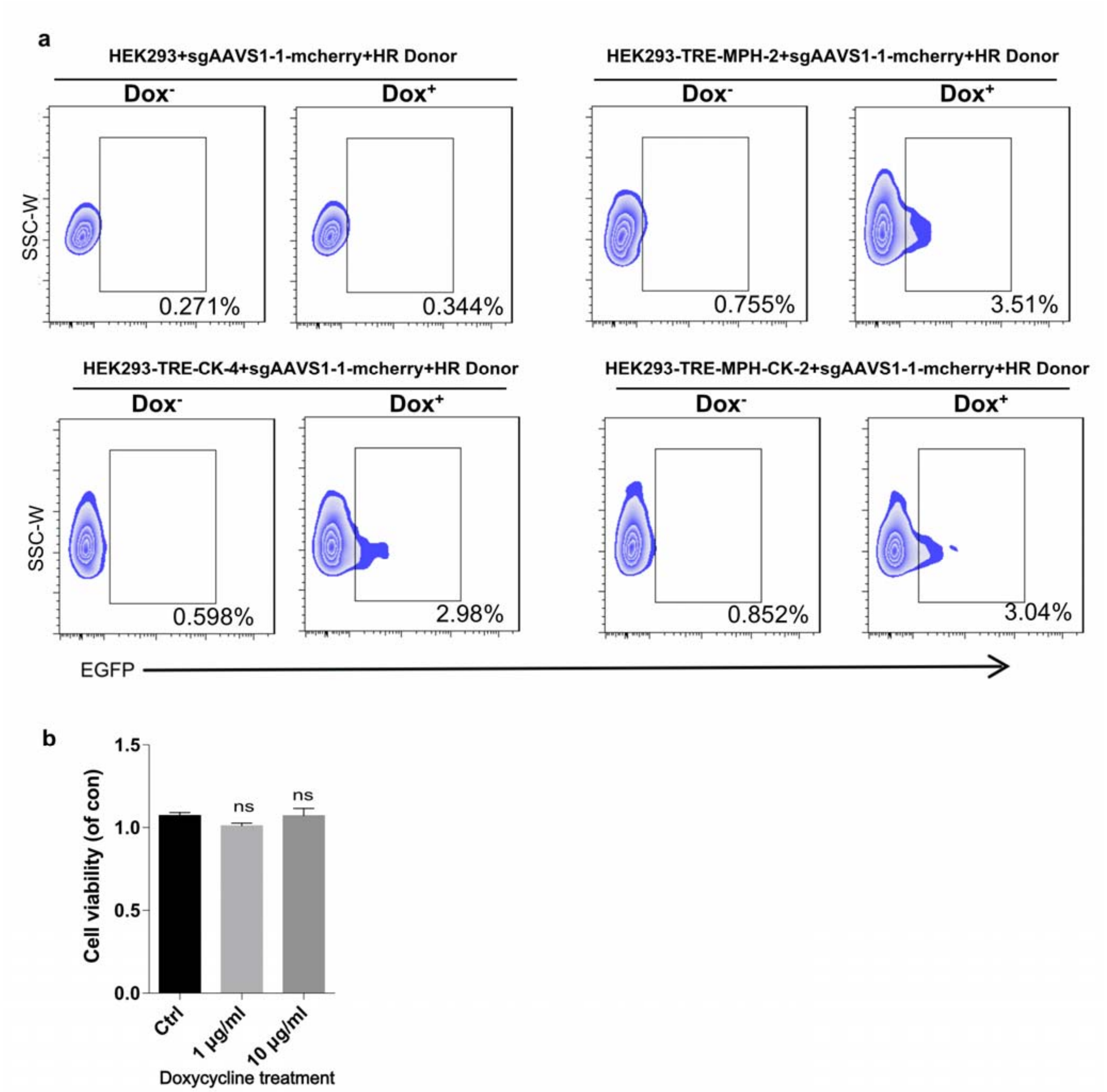

Supplementary Fig. S5 FACS plot for *AAVS1* targeting HDR enhancement using inducible CRISPRa/i system. (a) HEK293-TRE-MPH, HEK293-TRE-CK, and HEK293-TRE-MPH-CK cell lines were co-transfected with SA-T2A-EGFP donor and sgAAVS1-mCherry plasmid, 24 h later, 1 μg/ml doxycycline was provided. After 2 days' doxycycline treatment, the frequency of $EGFP^{+}$ cells within the population of $mCherry^{+}$ cells were analyzed by flow cytometry. **(b)** Cell viability was detected after Doxycycline treatment.

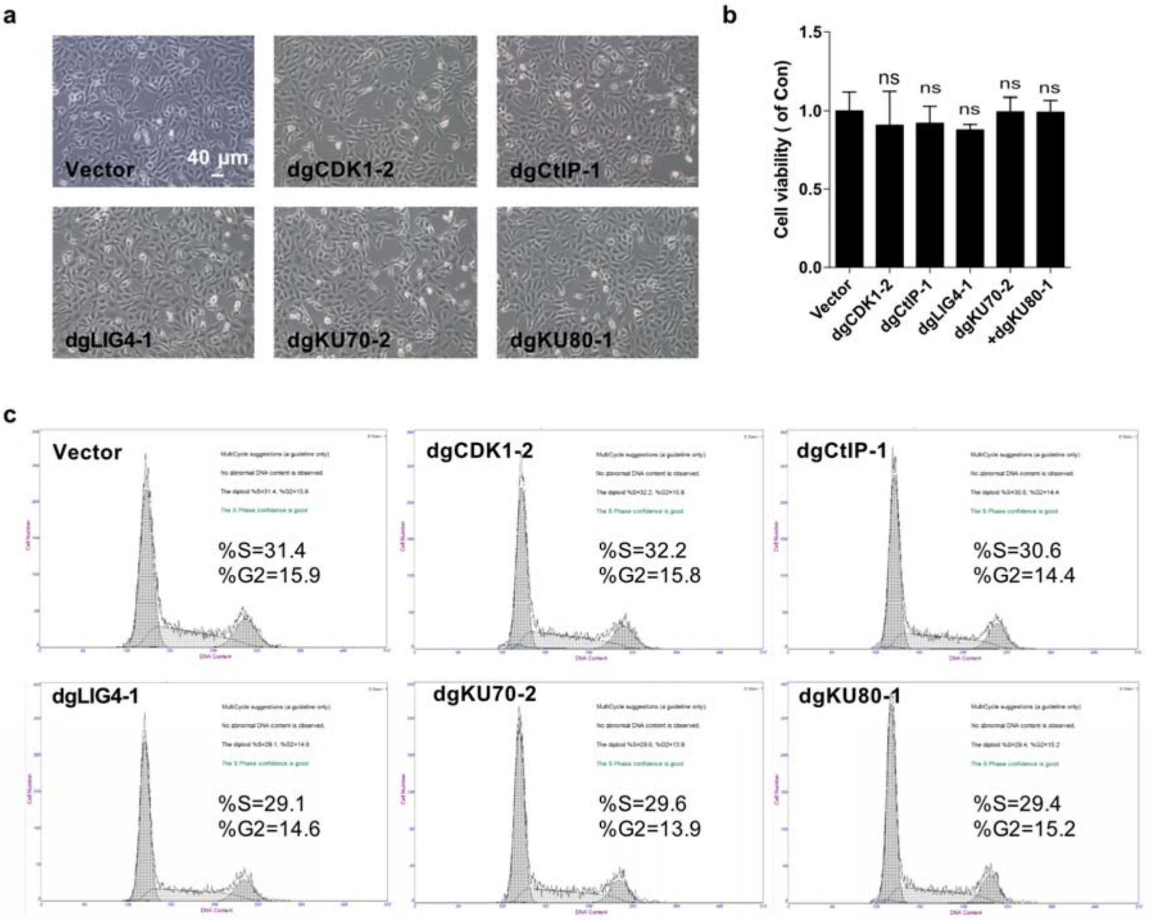

Supplementary Fig. S6 Cell viability and cell cycle confirmation after programming HDR and NHEJ pathways using CRISPRa/i system. (**a-b**) Cell viability was measured after doxycycline treatment. (**c**) Cell cycle was detected by Flow Cytometry after programming HDR and NHEJ pathways.

Programmable DNA Repair with CRISPRa/i

Enhanced Homology-Directed Repair Efficiency with a Single Cas9

Lupeng Ye[1,2,3,*], Chengkun Wang[1,*], Lingjuan Hong[1,4], Ninghe Sun[1], Danyang Chen[1], Sidi Chen[2,3,#], and Feng Han[1,#]

Supplementary Table S1 Target sequences of dgRNAs

Target gene	Name	Sequence (5'>3')
CDK1	dgCDK1-1	GCGCTCTAGCCACC
	dgCDK1-2	ACGGGCTACCCGAT
	dgCDK1-3	GCGCTCGCACTCAGT
	dgCDK1-4	CTAGTCAGCGGAGC
	dgCDK1-5	GAACTGTGCCAATGC
CtIP	dgCtIP-1	GCGTGACGTCGCGC
	dgCtIP-2	GGGCAGCTGGAGGAA
	dgCtIP-3	ATCGCCCTCCGGGAT
	dgCtIP-4	GTCGCCAGACTCTTC
	dgCtIP-5	GCATCAAGCCCTTG
Ligase IV	dgLIG4-1	GGCCCTTAAAACTT
	dgLIG4-2	ACACTTCAGTGCAC
	dgLIG4-3	TACCTCGGCGGCGT
	dgLIG4-4	GAGCCCCCGCGACGG
	dgLIG4-5	GGGGCTCACTGGCAG

KU70	dgKU70-1	GGTAGAAGCTGGTTG
	dgKU70-2	GTTGGCTTTCGTCA
KU80	dgKU80-1	GCATGCTCAGAGTTC
	dgKU80-2	GCCTTTCAGGCCTAGC
	dgKU80-3	GTACTAGCGTTTCAGG
ASCL1	dgASCL1	GCTCGCTGCAGCAG
HBG1	dgHBG1	GAGGCCAGGGGCCGG
EGFP	dgGFP-A1	ATTAGTCAGCAACC
EGFP	dgGFP-A2	ACTGGGCGGAGTTAG
EGFP	dgGFP-R1	GGCCGAGGCCGCCT
EGFP	dgGFP-R2	CAGAAGTAGTGAGG

Supplementary Table S2 Primers used for qRT-PCR

Target gene	Name	Sequence (5'>3')
GFP	GFP-qF	TGACCTACGGCGTGCAGTGCTT
	GFP-qR	CCTCGAACTTCACCTCGGCGC
GADPH	GADPH-qF	TTTGGTCGTATTGGGCGCCTGG
	GADPH-qR	CTCAGCCTTGACGGTGCCATGG
ASCL1	ASCL1-qF	GAGGAGCAGGAGCTTCTCGACT
	ASCL1-qR	AACGCCACTGACAAGAAAGCAC
HBG1	HBG1-qF	GGCTACTATCACAAGCCTGTGG
	HBG1-qR	TTGCCCATGATGGCAGAGGCA

CDK1	CDK1-qF	CTACAGGTCAAGTGGTAGCCATG
	CDK1-qR	CTGGAATCCTGCATAAGCACATCC
CtIP	CtIP-qF	CAACAGCTGAGGGAACAGCAG
	CtIP-qR	AGTTTAAGATCCTGCTGCCGG
Ligase IV	LIG4-qF	GGTAAAGGATCACGGGGTGG
	LIG4-qR	GCTGCTTGGTGGAGCTTTTC
KU70	KU70-qF	GGCTGTGGTGTTCTATGGTACCG
	KU70-qR	CCGTGGCCCATCATGTCTTGGA
KU80	KU80-qF	GTTGTGCTGTGTATGGACGTGG
	KU80-qR	GTGCCATCAGTACCAAACAGGAC

Supplementary Table S3 Primers for PCR amplification of sgRNA target region

Target gene	Name	Sequence (5’>3’)
EGFP	F	AGATCTATGGTGAGCAAGGGCGAGGA
	R	GAATTCTTACTTGTACAGCTCGTCCATG
AAVS1	Primer-F	GGGTCACCTCTACGGCTGG
	Primer-R	CGAATTCTTACTTGTACAGCTCGTCCA

Supplementary Table S4 Target sequences of sgRNAs

Target gene	Name	Sequence (5’>3’)
Venus	sgVenus	GAGCAGCGTCTTCGAGAGTG

AAVS1	sgAAVS1-1	CACCCCACAGTGGGGCCACT
AAVS1	sgAAVS1-2	TGTCCCTAGTGGCCCCACTG
ACTB	sgACTB	CCACCGCAAATGCTTCTAGG

Supplementary sequence 1. The TLR DNA sequence

Venus

CRISPR targeting site

T2A

mCherry

atggtgagcaagggcgaggagctgttcaccggggtggtgcccatcctggtcgagctggacggcgacg

taaacggccacaagttcagcgtgtccggcgagggcgagggcgatgccacctacggcaagctgaccct

gaagttcatctgcaccaccggcaacctgcagggagcagcgtcttcgagagtgaggacactagtgtgaa

ccctgacctacggcgtgcagtgcttcagccgctaccccgaccacatgaagcagcacgacttcttcaagt

ccgccatgcccgaaggctacgtccaggagcgcaccatcttcttcaaggacgacggcaactacaagacc

cgcgccgaggtgaagttcgagggcgacaccctggtgaaccgcatcgagctgaagggcatcgacttca

aggaggacggcaacatcctggggcacaagctggagtacaactacaacagccacaacgtctatatcatg

gccgacaagcagaagaacggcatcaaggtgaacttcaagatccgccacaacatcgaggacggcagc

gtgcagctcgccgaccactaccagcagaacacccccatcggcgacggccccgtgctgctgcccgaca

accactacctgagcacccagtccgccctgagcaaagaccccaacgagaagcgcgatcacatggtcct

gctggagttcgtgaccgccgccgggatcactctcggcatggacgagctgtacaagtaaGAATTCc

gGAGGGCAGAGGAAGTCTGCTAACATGCGGTGACGTCGAGGAG

AATCCTGGCCCAGGATCCgtgagcaagggcgaggaggataactccgccatcatcaag

gagttcctgcgcttcaaggtgcacatggagggctccgtgaacggccacgagttcgagatcgagggcga

gggcgagggccgcccctacgagggcacccagaccgccaagctgaaggtgaccaagggtggcccc

tgcccttcgcctgggacatcctgtcccctcagttcatgtacggctccaaggcctacgtgaagcaccccgc

cgacatccccgactacttgaagctgtccttccccgagggcttcaagtgggagcgcgtgatgaacttcga

ggacggcggcgtggtgaccgtgacccaggactcctctctgcaggacggcgagttcatctacaaggtga

agctgcgcggcaccaacttcccctccgacggccccgtaatgcagaagaagaccatgggctgggaggc

ctcctccgagcggatgtaccccgaggacggcgccctgaagggcgagatcaagcagaggctgaagct

gaaggacggcggccactacgacgctgaggtcaagaccacctacaaggccaagaagcccgtgcagct

gcccggcgcctacaacgtcaacatcaagttggacatcacctcccacaacgaggactacaccatcgtgg

aacagtacgaacgcgccgagggccgccactccaccggcggcatggacgagctgtacaagtga

Supplementary sequence 2. The AAVS1 HDR donor DNA sequence

Left homology arm of *AAVS1*

SA-T2A-EGFP-ShortPA

Right homology arm of *AAVS1*

5’-ttctccttctggggcctgtgccatctctcgtttcttaggatggccttctccgacggatgtctcccttgcgt

cccgcctccccttcttgtaggcctgcatcatcaccgtttttctggacaaccccaaagtaccccgtctccctg

gctttagccacctctccatcctcttgctttctttgcctggacaccccgttctcctgtggattcgggtcacctct

cactcctttcatttgggcagctcccctaccccccttacctctctagtctgtgctagctcttccagccccctgtc

atggcatcttccaggggtccgagagctcagctagtcttcttcctccaacccgggcccctatgtccacttca

ggacagcatgtttgctgcctccagggatcctgtgtccccgagctgggaccaccttatattcccagggccg

gttaatgtggctctggttctgggtacttttatctgtcccctccaccccacagtgggg ggtacc agtcgatcc

aacatggcgacttgtcccatccccggcatgtttaaatatactaattattcttgaactaattttaatcaaccgatt

tatctctcttccgcaggtggcggaggttccggtggaagcggaggtagcggcggatccgagggccgcg

gcagcctgctgacctgcggcgatgtggaggagaaccccgggcccATGGTGAGCAAGGG

CGAGGAGCTGTTCACCGGGGTGGTGCCCATCCTGGTCGAGCTG

GACGGCGACGTAAACGGCCACAAGTTCAGCGTGTCCGGCGAG

GGCGAGGGCGATGCCACCTACGGCAAGCTGACCCTGAAGTTCA

TCTGCACCACCGGCAAGCTGCCCGTGCCCTGGCCCACCCTCGT

GACCACCCTGACCTACGGCGTGCAGTGCTTCAGCCGCTACCCC

GACCACATGAAGCAGCACGACTTCTTCAAGTCCGCCATGCCCG

AAGGCTACGTCCAGGAGCGCACCATCTTCTTCAAGGACGACGG

CAACTACAAGACCCGCGCCGAGGTGAAGTTCGAGGGCGACAC

CCTGGTGAACCGCATCGAGCTGAAGGGCATCGACTTCAAGGAG

GACGGCAACATCCTGGGGCACAAGCTGGAGTACAACTACAACA

GCCACAACGTCTATATCATGGCCGACAAGCAGAAGAACGGCAT

CAAGGTGAACTTCAAGATCCGCCACAACATCGAGGACGGCAGC

GTGCAGCTCGCCGACCACTACCAGCAGAACACCCCCATCGGCG

ACGGCCCCGTGCTGCTGCCCGACAACCACTACCTGAGCACCCA

GTCCGCCCTGAGCAAAGACCCCAACGAGAAGCGCGATCACATG

GTCCTGCTGGAGTTCGTGACCGCCGCCGGGATCACTCTCGGCAT

GGACGAGCTGTACAAGTAAAATAAAAGATCTTTATTTTCATTAG
ATCTGTGTGTTGGTTTTTTGTGTgaattcccactagggacaggattggtgacagaaa
agccccatccttaggcctcctccttcctagtctcctgatattgggtctaacccccacctcctgttaggcagat
tccttatctggtgacacacccccatttcctggagccatctctctccttgccagaacctctaaggtttgcttac
gatggagccagagaggatcctgggagggagagcttggcaggggggtgggagggaagggggggatg
cgtgacctgcccggttctcagtggccaccctgcgctaccctctcccagaacctgagctgctctgacgcg
gctgtctggtgcgtttcactgatcctggtgctgcagcttccttacacttcccaagaggagaagcagtttgga
aaaacaaaatcagaataagttggtcctgagttctaactttggctcttcacctttctagtccccaatttatattgtt
cctccgtgcgtcagttttacctgtgagataaggccagtagccagccccgtcctggcagggctgtggtga
ggagggggtgtccgtgtggaaaactccctttgtgagaatggtgcgtcctaggtgttcaccaggtcgtgg
ccgcctctactccctttctctttctccatccttctttccttaaagagtccccagtgctatctgggacatattcctc
cgcccagagcagggtcccgcttccctaaggccctgctctgggcttctgggtttgagtccttggcaagccc
aggagaggcgctcaggcttccctgtccccttcctcgtccaccatctcatgcccctggctctcctgcccct
tccctacaggggttcctggctctgctcttcagactgagccccgt-3’

Supplementary sequence 3. The ACTB HDR donor DNA sequence

Left homology arm of *ACTB*

T2A-EGFP

Right homology arm of *ACTB*

5’-CGGCTCTGCCTGACATGAGGGTTACCCCTCGGGGCTGTGCTG
TGGAAGCTAAGTCCTGCCCTCATTTCCCTCTCAGGCATGGAGTC
CTGTGGCATCCACGAAACTACCTTCAACTCCATCATGAAGTGTG

ACGTGGACATCCGCAAAGACCTGTACGCCAACACAGTGCTGTC
TGGCGGCACCACCATGTACCCTGGCATTGCCGACAGGATGCAG
AAGGAGATCACTGCCCTGGCACCCAGCACAATGAAGATCAAGG
TGGGTGTCTTTCCTGCCTGAGCTGACCTGGGCAGGTCGGCTGT
GGGGTCCTGTGGTGTGTGGGGAGCTGTCACATCCAGGGTCCTC
ACTGCCTGTCCCCTTCCCTCCTCAGATCATTGCTCCTCCTGAGC
GCAAGTACTCCGTGTGGATCGGCGGCTCCATCCTGGCCTCGCTG
TCCACCTTCCAGCAGATGTGGATCAGCAAGCAGGAGTATGACG
AGTCCGGCCCCTCCATCGTCCACCGCAAATGCTTCgagggccgcggca
gcctgctgacctgcggcgatgtggaggagaaccccgggcccATGGTGAGCAAGGGCG
AGGAGCTGTTCACCGGGGTGGTGCCCATCCTGGTCGAGCTGGA
CGGCGACGTAAACGGCCACAAGTTCAGCGTGTCCGGCGAGGG
CGAGGGCGATGCCACCTACGGCAAGCTGACCCTGAAGTTCATC
TGCACCACCGGCAAGCTGCCCGTGCCCTGGCCCACCCTCGTGA
CCACCCTGACCTACGGCGTGCAGTGCTTCAGCCGCTACCCCGA
CCACATGAAGCAGCACGACTTCTTCAAGTCCGCCATGCCCGAA
GGCTACGTCCAGGAGCGCACCATCTTCTTCAAGGACGACGGCA
ACTACAAGACCCGCGCCGAGGTGAAGTTCGAGGGCGACACCCT
GGTGAACCGCATCGAGCTGAAGGGCATCGACTTCAAGGAGGAC
GGCAACATCCTGGGGCACAAGCTGGAGTACAACTACAACAGCC
ACAACGTCTATATCATGGCCGACAAGCAGAAGAACGGCATCAA
GGTGAACTTCAAGATCCGCCACAACATCGAGGACGGCAGCGTG

CAGCTCGCCGACCACTACCAGCAGAACACCCCCATCGGCGACG

GCCCCGTGCTGCTGCCCGACAACCACTACCTGAGCACCCAGTC

CGCCCTGAGCAAAGACCCCAACGAGAAGCGCGATCACATGGTC

CTGCTGGAGTTCGTGACCGCCGCCGGGATCACTCTCGGCATGG

ACGAGCTGTACAAGTAAtaggcggactatgacttagttgcgttacaccctttcttgacaaa

acctaacttgcgcagaaaacaagatgagattggcatggctttatttgttttttttgttttgttttggtttttttttttttt

ttggcttgactcaggatttaaaaactggaacggtgaaggtgacagcagtcggttggagcgagcatcccc

caaagttcacaatgtggccgaggactttgattgcacattgttgtttttttaatagtcattccaaatatgagatgc

gttgttacaggaagtcccttgccatcctaaaagccaccccacttctctctaaggagaatggcccagtcctc

tcccaagtccacacaggggaggtgatagcattgctttcgtgtaaattatgtaatgcaaaatttttttaatcttc

gccttaatactttttttattttgttttattttgaatgatgagccttcgtgcccccccttcccccttttttgtcccccaa

cttgagatgtatgaaggcttttggtctccctgggagtgggtggaggcagccagggcttacctgtacactg

acttgagaccagttgaataaaagtgcacaccttaaaaatgaggccaagtgtgactttgtggtgtggctgg

gttgggggcagcagagggtgaaccctgcaggagggtgaaccctgcaaaagggtggggcagtgggg

gccaacttgtccttacccagagtgcaggtgtgtggagatccctcctgccttgacattgagcagccttagag

ggtgggggaggctcaggggtcaggtctctgttcctgcttattgggga-3’
